# Women’s recall of maternal and newborn interventions received in the postnatal period: a validity study in Kenya and Swaziland

**DOI:** 10.7189/jogh.08.010605

**Published:** 2018-06

**Authors:** Katharine J McCarthy, Ann K Blanc, Charlotte E Warren, Brian Mdawida

**Affiliations:** 1 Population Council, New York, New York, USA; 2 Population Council, Washington, D.C., USA; 3 Population Service Kenya, Nairobi, Kenya

## Abstract

**Background:**

Despite the concentration of maternal and infant deaths in the early postnatal period, information on the content and quality of postnatal care interventions is not routinely collected in most low and middle-income countries. At present, data on the coverage of postnatal care interventions mostly rely on women’s reports collected in household surveys, such as the Demographic and Health Surveys (DHS) and Multiple Indicator Cluster Surveys (MICS), which collect limited information. We assessed the validity of a set of postnatal care indicators that reflect a range of recommended interventions for both mother and newborn and have potential to be included in household surveys for monitoring of population-level coverage.

**Methods:**

We compared women’s reports in exit interviews on the content of postnatal care received in health facilities located in Kenya and Swaziland against a gold standard of direct observation by a trained third party. We calculated sensitivity, specificity and the area under the receiver operating curve (AUC) to assess individual-level reporting accuracy and the inflation factor (IF) to assess population-level accuracy. We also examined whether women’s reporting accuracy varied significantly by her sociodemographic characteristics.

**Results:**

18 indicators in Kenya and 19 in Swaziland had sufficient sample size for analysis. Of these, 12 indicators in Kenya and five in Swaziland met criteria for acceptable individual and population-level reporting accuracy. Two indicators met acceptability criteria in both Kenya and Swaziland: whether the provider performed a breast exam or an abdominal exam. There was no significant association between women’s characteristics and reporting accuracy, across indicators.

**Conclusion:**

Women are able to accurately report on multiple aspects of care received during a postnatal visit. Findings inform the recommendation of indicators for tracking progress of critical postnatal care interventions for mothers and newborns. Improved measurement of the coverage of maternal and newborn postnatal care is warranted to monitor progress in maternal and newborn care globally.

More maternal deaths occur in the time period following the first 24 hours of birth and within 6 weeks of delivery (36%) than any other phase of pregnancy and childbirth [1]. Hemorrhage, the leading cause of maternal death (27%), most often occurs in the postnatal period as does sepsis, which accounts for an additional 11% of maternal deaths [2,3]. The postnatal period is also a high-risk period for child health. Approximately two in five (45%) child deaths under age five occur within the first 28 days of birth [4]. Furthermore, the initiation of health behaviors such as breastfeeding, immunization visits and the use of postnatal HIV services in the first six weeks of life have lasting effects on development [5,6]. The potential benefit of early detection and delivery of a range of interventions is the basis for recommendations that postnatal health checks occur within the critical first two days of birth.

In low and middle-income country (LMIC) settings, where the vast majority of maternal and newborn deaths occur, data on the coverage of postnatal care interventions often rely on women’s reports collected in household surveys, such as the Demographic and Health Surveys (DHS) and Multiple Indicator Cluster Surveys (MICS). At present, these survey programs collect limited information on care received in the postnatal period. Current questionnaires (Round 7 DHS and MICS-5) each measure whether a mother or newborn had contact with a facility or provider during the postnatal period. However, implicit in reliance on these general contact indicators is the assumption that women who receive facility-based postnatal care or are visited by a provider will also receive key interventions. Several researchers have highlighted discrepancies between contact with care and receiving quality care [7-9]. Measuring the interventions that a woman actually receives is more informative than measuring contact with care, provided that women can accurately report this information.

In response, various groups have sought to identify and add standard measures related to key maternal and newborn health interventions in the postnatal period to monitoring systems [10,11]. The most recent DHS (Round 7) now includes one question on the content of postnatal care received within the first two days of birth. This question asks, “*During the first two days after (NAMES)’s birth, did any health care provider do the following: examine the cord, measure the infant’s temperature, counsel mother on danger signs for newborns, counsel mother on breastfeeding, observe breastfeeding*” [12]. Other related questions include early initiation of breastfeeding (within one hour of birth), whether the newborn was placed skin-to-skin with the mother, whether the baby was weighed at the time of birth, and receipt of child immunizations. Additionally, an optional module on Pregnancy and Postnatal Care is currently available for inclusion in the DHS and MICS.

The WHO has issued 12 recommendations related to the provision of care in the postnatal period for both mother and newborn, nine of which focus on content of care [13]. At present, however, no questions in the DHS or MICS relate to the content of postnatal interventions received by the mother. The content of maternal and newborn care delivered in the period following the first 2 days of birth is also not routinely tracked within health management information systems.

Furthermore, evidence gaps remain with regard to how accurately women can report on postnatal interventions. We identified only one study in rural China that attempted to validate the accuracy of women’s recall of postnatal care interventions received in the six weeks following delivery using quantitative methods [14]. This study compared women’s reports of maternal postnatal care and child immunizations against facility medical records. Use of facility records as the reference standard, however, somewhat limits the study findings as records may be subject to incomplete or inaccurate reporting. In addition, two qualitative studies which assessed postnatal care practices in Ghana [15] and in Bangladesh and Malawi [16], found women had difficulty understanding questions related to postnatal care contact. Phrases such as whether the woman received a “health checkup” or “check on your health” in this period required additional clarification by interviewers [10,16].

The present study addresses these gaps in the evidence base by assessing the validity of a set of postnatal care indicators that reflect a range of recommended PNC intervention and counseling procedures. Our research question is: can women accurately report on the content of postnatal care received at a health facility? We compare women’s reports of postnatal care received against observations by a trained third party observer using a structured checklist in health facilities located in Kenya and Swaziland. Findings inform the recommendation of indicators for tracking progress as well as strategies needed to enhance the monitoring of critical postnatal care interventions for mothers and newborns.

## METHODS

### Data sources

We conducted secondary analysis of previously collected, de-identified facility-based data to compare women’s reports of postnatal care received against observations by trained third party observers using a structured checklist in health facilities located in Kenya and Swaziland. Women’s reports of care received were collected via an exit interview conducted prior to her leaving the facility following a postnatal care visit between 24 hours and 10 weeks of birth. Data were initially collected as part of the Integra Initiative, a SRH/HIV integration intervention implemented by Population Council and the London School of Hygiene and Tropical Medicine. The full study protocol has previously been published [17].

The present analysis combines three rounds of cross-sectional data, collected in 2009, 2011 and 2012 in each country.

### Study population

The study population was comprised of women who attended a postnatal check for themselves and/or for their newborn at a participating study facility. Eligible women were: aged 15 years and older, a client attending a postnatal check for herself and/or her newborn (>24 hours to <10 weeks), lived in the catchment area of the health facility, and provided informed consent to be interviewed.

### Study locations

Client exit interviews and observations of postnatal care were conducted in 20 public health facilities located in Eastern province in Kenya and in three regions (Lubombo, Manzini and Shiselweni) in Swaziland. There were 12 participating study facilities in Kenya (4 hospitals and 8 health centers), and 8 in Swaziland (public health units/MCH-FP).

Study facilities in each country had participated in a sexual and reproductive health and HIV care integration study or were comparable to participating facilities using pair-wise matching (see Warren et al., 2012 for full details) [17]. All facilities had high client load (>50 infants/mo receiving their first immunizations at 6 weeks at the postnatal care (PNC)-HIV clinics), a minimum of two providers qualified in and currently delivering family planning services, and provided a range of services including counseling and provision of family planning, voluntary counseling and testing, STI treatment, and interventions related to the prevention of mother-to-child HIV transmission.

At the time data were collected, facilities located in Eastern province, Kenya, served populations in which approximately 56% of women aged 15-49 who had a live birth in the preceding five years had received a postnatal check-up. In 2014, 53% of Kenyan women nationally who had a live birth in the preceding two years received postnatal care within the recommended two days following birth, an increase from 42% in 2008 [18]. The most recent data available for Swaziland illustrate that 25% of reproductive-aged women who had a live birth in the preceding five years received a postnatal check, while 22% of women received a check within the first two days of birth [19]. In Swaziland, there was little variation in postnatal coverage by region.

### Data collection

Each postnatal care (PNC) client aged 15 years and over attending a consultation on the day of the research team’s visit to the facility was invited to participate in the study until the desired sample size was reached. In both countries, at least 16 postpartum women were observed per study facility for each round of data collection. All eligible women were provided with a brief description of the study. If the client was willing to participate, her written informed consent to be interviewed and observed was obtained prior to the start of the visit. Each observed client was interviewed immediately after the PNC consultation to measure perceptions of the services received.

Observations of the provision of postnatal care were conducted by a trained third party using a structured checklist. Observations included both provider-client interactions (ie, how clients were treated and whether they actively participated), and the technical content of provided care. All health care providers who provide postnatal care services in the study facilities were invited to participate in the study at the time of data collection. If providers agreed to participate, their informed consent was obtained prior to observation. To reduce the risk of biasing client provider interactions in the positive direction, more than one day of observations were conducted at each facility to normalize the presence of the observer.

Data collectors who administered the exit interviews were diploma/degree holders in a social science and were not the same individuals as researchers selected to observe postnatal consultations. Client exit interviews were conducted in places where women had visual and auditory privacy to ensure confidentiality. Study observers were qualified nurse/midwives who were either retired, newly qualified, or from facilities outside the research sites. Observers were trained by the research team to be non-participant observers of the provider, client, and newborn PNC-related interactions. All data collectors were trained in ethical research and fluent in the local language spoken in the facility.

### Questionnaires

The Integra Initiative aimed to strengthen provider capacity to provide postnatal care for the (1) infant and (2) the mother, integrated with (3) family planning, (4) HIV counseling, testing and services, and (5) screening/management for sexually transmitted infections [17]. We attempted to validate all indicators related to these five areas for which there was a comparable client exit interview question and observation record. The correspondence of assessed indicators to PNC recommendations as issued by global health agencies and initiatives are indicated in **Table 1**.

**Table 1:** Postnatal care indicators assessed in study and inclusion in global health initiatives, by round of data collection and country

			Question by round of data Collection*		Sufficient N?†	
**Indicator**	**Client Exit Interview Question**	**Inclusion in WHO Guidelines for PNC‡**	**Kenya (KY)**	**Swaziland (SZ)**	**Kenya (KY)**	**Swaziland (SZ)**
	During your visit today, did the provider:					
Blood pressure check	Measure your blood pressure?	WHO: Recommended as part of well-being assessment (Rec. No. 8), detect and manage eclampsia	R2, R3	R2, R3	Y	Y
Breast exam	Examine your breasts?	WHO: Assessment of breast pain at each postnatal contact beyond 24 h of birth (Rec. No. 8), avoid breast infection	R2, R3	R2, R3	Y	Y
Examine abdomen	Examine your abdomen?	WHO: Assessment of uterine tenderness at each postnatal contact 24 h of birth (Rec. No. 8), avoid infection	R2, R3	R2, R3	Y	Y
Examine vagina	Examine your vagina?	WHO: Assessment of perineal wound healing at each postnatal contact beyond 24 h of birth (Rec. No. 8), avoid infection	R2, R3	R2, R3	Y	Y
Screen for cervical cancer	Check you for cervical cancer?		R2, R3	R2, R3	N	Y
Check anemia (pallor or refer to HB test)	Check you for anemia?	WHO: Detect and treat anemia, iron supplementation (Rec. No. 10)	R2, R3	R2, R3	Y	Y
Contact with Nurse or Nurse/Midwife	Who attended to you?	WHO: Recommended as part of well-being assessment (Rec. No. 8)	R2, R3	R2, R3	N	N
Contact with Doctor	Who attended to you?		R2, R3	R2, R3	N	N
Ask about excessive bleeding	Ask if you had any abnormal bleeding?	WHO: Recommended as part of well-being assessment (Rec No. 8), prevent infection and hemorrhage	R2, R3	R2, R3	Y	Y
Discuss danger signs after birth	Discuss with you danger signs after birth?	WHO: Counseling on signs and symptoms of: postpartum hemorrhage, pre-eclampsia/eclampsia, infection, thromboembolism (Rec. No. 9)	R2, R3	R2, R3	Y	Y
Discuss how soon after delivery a woman can get pregnant	Did a health provider tell you how soon after delivery a woman can get pregnant?		R0, R2, R3	R0, R2, R3	Y	Y
Discuss return to fertility	Did the provider discuss return to facility?	WHO: Counseling on birth spacing and family planning (Rec. No. 9)	R0, R2, R3	R0, R2, R3	Y	Y
Discuss benefits of birth spacing	Did any health provider talk to you about the importance of waiting some time before getting pregnant again?	WHO: Counseling on birth spacing and family planning (Rec. No. 9)	R0, R2, R3	R0, R2, R3	Y	Y
Discuss return to sexual activity	Did the provider discuss return to sexual activity?	WHO: Counseling on resumption of sexual intercourse two to six weeks after birth (Rec. No. 8)	R0, R2, R3	R0, R2, R3	Y	Y
Discussed a FP method (incl. natural)	Did the provider discuss with you family planning? (KY: R2, R3, SZ: R2, R3) During your time in this facility, did you receive any information about family planning methods? (KY: R0, SZ: R0)	WHO: Counseling on contraceptive options, contraceptive methods should be provided if requested (Rec. No. 9)	R0, R2, R3	R2, R3	Y	Y
Received any modern FP method	Which family planning method(s) did you receive today?		R2, R3	R0, R2, R3	N	Y
Discuss how chosen FP method works	For the method(s) you received today, did the provider discuss with you how the method works?		R0, R2, R3	R0, R2, R3	N	N
Explains advan/disad of chosen FP method	For the method(s) you received today, did the provider explain the advantages/disadvantages of the method?		R0, R2, R3	R0, R2, R3	N	Y
Discussed STIs or HIV/AIDS	Did the provider give you information or advice on sexually transmitted infections or the AIDS virus?	WHO: Counseling on safer sex including use of condoms (Rec. No. 9), prevent and identify STIs and HIV	R2, R3	R2, R3	Y	Y
Discuss breastfeeding/feeding for baby	Did any health provider discuss breastfeeding/feeding for the baby?	WHO: Counseling and support for exclusive breastfeeding at each postnatal contact (Rec. No. 5)	R0, R2, R3	R0, R2, R3	Y	N
Examine baby (undressed)	Did the provider examine the baby (physical check, unclothed)?	WHO: Assessment at each postnatal contact for newborn should include signs such as fever, low body temperature, jaundice or yellow palms and soles, fast breathing, severe chest in-drawing or no spontaneous movement, occurs (Rec. No. 4)	R2, R3	R2, R3	Y	Y
Weigh the baby	Did the provider weigh the baby?	WHO: Low birth weight babies should be identified immediately as provided special care per existing WHO guidelines (Rec. No. 7)	R2, R3	R2, R3	Y	N
Immunize baby§	Did the provider immunize the baby?	WHO: Immunization should be promoted as per existing WHO guidelines (Rec. No. 7)	R0, R2, R3	R0, R2, R3	Y	Y
Gave information on baby’s sickness signs§	Did the provider give you information on the baby's sickness signs? (KY | SZ, R2, R3) Did any provider tell you about danger signs that you should look out for in the baby? (KY|SZ, R0)	WHO: Assessment at each postnatal contact for newborn should include danger signs (eg, high or low body temperature, jaundice or yellow palms and soles, abnormal respiration (Rec. No. 4)	R0, R2, R3	R0, R2, R3	Y	Y

*R0, R2 and R3 represents data collection rounds for 2009, 2011, and 2012, respectively.

†Sufficient sample size refers to having at least 5 counts per cell of two-by-two tables constructed for observer vs women’s report of whether intervention was received (Y/N).

‡WHO Recommended Postnatal Interventions for the Mother and Newborn (WHO, 2013).

§Aspect of care also measured in DHS (Available at: http://www.dhsprogram.comand/) and/or MICS surveys (Available at: http://www.unicef.org/statistics/index_24302.html).

### Ethical clearance

Ethical clearance for the Integra protocol was granted by the Population Council’s Institutional Review Board (IRB) (approval number 444), the Ethics Review Committee of London School of Hygiene & Tropical Medicine (approval number 5426), the Kenya Medical Research Institute (KEMRI) Ethical Review Board (approval number 114), and the Scientific Ethics Committee of the Swaziland Ministry of Health (approval number MH/599C). For the present study, an exemption waiver from the Population Council IRB was obtained to conduct secondary analysis of the de-identified data. No analysis took place prior to receiving the exemption waiver.

### Data management and analysis

For each participant, a unique identification code for the client exit interview and observation record of received postnatal care were matched. Identification codes were generated by combining information on the facility, date of interview, start/end time of observation, the age of the baby (in weeks), and the type of visit (eg, whether was for PNC). Cases with missing or incomplete data were excluded. Given the number of variables used and relatively few observations per facility each day (N<20), we are confident in the accuracy of the matching process.

Data for each cross-sectional year of data collection were pooled for each country. Questions about whether interventions occurred were coded one if the response was “Yes” and all other responses were coded as zero.

### Sample size

Sample size was estimated using pooled rounds of cross-sectional data for each country. We anticipated indicator prevalence would range between 50 and 80% coverage, as assessed indicators were health promoting only (rather than harmful practices). We assumed levels of moderate to high sensitivity (60 to 70%) and specificity (70 to 80%), given the short duration of recall (women were interviewed immediately following the PNC consultation). Sample size for anticipated sensitivity and specificity levels was calculated using Buderer’s formula [20]. We set α = 0.05 for both accuracy parameters assuming a normal approximation to a binomial distribution. Based on these specifications, a sample size of 400 women per country is sufficient to estimate 60% sensitivity and 70% specificity with at least 7% precision.

### Statistical analysis

Estimates of sensitivity, specificity for each indicator were calculated in R Studio (Version 3.3.1; Boston, USA). The pROC package was used to obtain area under the receiver operating curve (AUC) estimates using nonparametric analysis [21]. Confidence intervals were calculated assuming a binomial distribution.

Receiver operating curve analysis is a valuable method to describe the accuracy of diagnostic tools by plotting the tradeoff between sensitivity (true positive rate) against its false positive rate (1 – specificity). In practice, the AUC represents the “average accuracy of a diagnostic test” and summarizes the accuracy in a single number [22-24]. AUC values can range from zero to one, with an AUC of 1.0 representing perfect diagnostic accuracy, while an AUC of 0.5 represents a random response [24]. AUC can be considered a measure of individual level accuracy of reporting. Study acceptability criteria for a “valid indicator” were set a priori at AUC<0.60 as low accuracy, 0.60≤AUC≤0.70 as moderate accuracy, and AUC>0.70 as high accuracy. For the present analysis, an AUC of greater than 0.70 was considered acceptable.

To assess the population-based validity of an indicator, we also estimated the prevalence that would be obtained in a survey given its sensitivity and specificity (Pr). Each indicator’s estimated sensitivity (SE) and specificity (SP) was applied to its true prevalence (P) (ie, observer reported prevalence) using the following equation: Pr = P × (SE+SP – 1) + (1 – SP) [25]. The inflation factor (IF) is the ratio of the estimated survey-based prevalence to its true population prevalence (observer report) and represents the degree to which each indicator would be over- or under- estimated if assessed using a population-based survey [26]. Study acceptability criteria for IF was between 0.75 and 1.25, and is informed by criteria previously applied in the literature [27,28].

**Figure 1:**
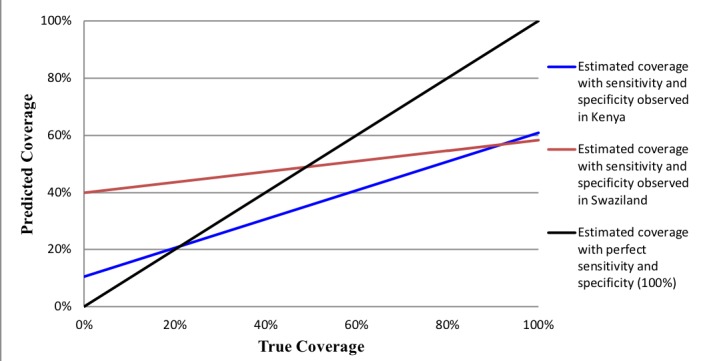
Predicted coverage of whether the provider discussed danger signs for the mother after birth in Kenya and Swaziland based on sensitivity and specificity of women's recall across all possible levels of true coverage.

**Figure 2:**
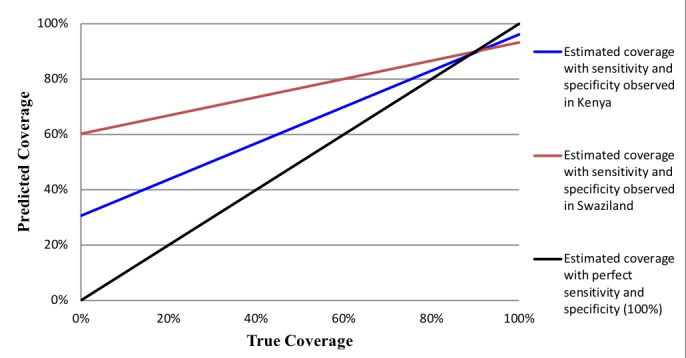
Predicted coverage of infant immunization in Kenya and Swaziland based on sensitivity and specificity of women's recall across all possible levels of true coverage.

The properties of the estimated survey-based prevalence are that when an intervention’s observed prevalence is high, the IF ratio will approximate sensitivity irrespective of specificity [14,26]. The implications of this relationship for population-level validity are that when the observed prevalence and sensitivity are high, the IF ratio will approximate 1, which is indicative of low bias. High coverage indicators may lack sufficient sample size to adequately measure specificity. With a low coverage indicator, a moderate false-positive rate will result in low specificity and produce a large IF, indicative of a biased measure. We caution against the generalization of the population-based validity assessments made in this study to other contexts with varying levels of coverage. **Figure 1** and **Figure 2** provide examples of how indicator properties from this study can be applied to settings with varying levels of intervention coverage. The coverage of indicators measured in this study is presented in the results.

### Covariate analysis

We stratified estimates of sensitivity, specificity and the AUC by covariates hypothesized to influence women’s reporting ability. These were: educational attainment, number of prior births, age of the baby (in weeks), age of the client, facility and survey year. Using methodology proposed by Janes, we modeled each indicator’s ROC curve to determine whether the addition of covariates significantly influenced discrimination accuracy [29]. Significance was determined using the Wald Test for each covariate. To account for correlation in the observed coverage of interventions within each facility, ROC regression models were adjusted for clustering using bootstrapping to obtain standard errors using the facility as the resampling unit. Covariate analysis was performed using the *rocreg* function in Stata Version 14 (College Station, TX, USA).

Outcomes were modeled for four variables: three were selected due to their inclusion in the DHS or MICS, whether the provider: (1) discussed breastfeeding/ feeding for the baby, (2) weighed the baby, or (3) immunized the baby. We assessed an additional indicator of postnatal care for the mother (4) – whether the provider discussed danger signs after birth for the mother. We selected this indicator given the implications for identifying and treating complications in the postnatal period for reducing maternal mortality. Currently no content of care indicators related to postnatal care for the mother are included in the DHS or MICS. Given the low sample size in Swaziland, covariate analysis was performed in the Kenya sample only.

## RESULTS

In total 1291 women were interviewed and observed (n = 646 in Kenya and 645 in Swaziland). Due to incomplete or missing data, it was possible to match 545 of 646 (84%) of cases in Kenya and 319 of 645 (50%) of cases in Swaziland. Some indicators were not measured in the first round of data collection in both countries but were included in later data collection rounds due to modifications to the questionnaire (**Table 1**). As a result, the sample size for some indicators was lower than anticipated.

### Sample description

Demographic characteristics among women in both country samples are shown in **Table 2**. Women’s age ranged from 15 to 44 years, the mean age for mothers in Kenya was 26.3 years (standard deviation, SD = 5.8) compared to 25.1 years for Swaziland (SD = 5.6). Infant age ranged from zero to ten weeks. Overall, women in Kenya were more likely to be married (86%) compared to less than half (45%) in Swaziland. The majority of women in Swaziland had completed secondary school or higher (74%), relative to 19% of Kenyan women.

**Table 2:** Sample characteristics by country

	Kenya	Swaziland
**N (%)**	**N (%)**
**N = 545**	**N = 319**
**Round:**		
R0 2009	221 (40.5)	193 (60.5)
R2 2011	127 (23.3)	72 (22.6)
R3 2012	198 (36.3)	54 (16.9)
**Age of client:**		
15-19	46 (8.6)	47 (14.7)
20-24	192 (35.7)	128 (40.1)
25-29	151 (28.1)	67 (21.0)
30-34	90 (16.7)	54 (16.9)
35-39	47 (8.7)	20 (6.3)
40-45	12 (2.2)	3 (0.9)
**Age of baby:**		
<2 weeks	116 (21.3)	52 (16.6)
2-4 weeks	121 (22.2)	10 (3.2)
5-6 weeks	240 (44.0)	229 (73.2)
7-10 weeks	68 (12.5)	22 (7.0)
**Marital status:**		
Never married	63 (11.6)	176 (55.3)
Married/live together	468 (86.0)	142 (44.7)
Separated/divorced/widowed	13 (2.4)	0 (0.0)
**Prior parity:**		
1	160 (29.9)	108 (34.2)
2	124 (23.2)	80 (25.3)
3	103 (19.3)	65 (20.6)
4+	148 (27.7)	63 (19.9)
**Education level:**		
Less than primary	217 (39.8)	24 (7.5)
Primary	224 (41.1)	59 (18.5)
Secondary or more	104 (19.1)	236 (74.0)

### Validation results

The assessment of validity was limited to those indicators with at least 5 cases in each cell of a two-by-two table that cross-tabulated the woman’s response (Yes, No) with the observer’s response (Yes, No) to calculate sensitivity and specificity. It was possible to validate women’s reporting on a range of aspects of the postnatal care visit in at least one country. The phases of the consultation we were able to assess include: physical examination of the mother (n = 6 indicators), advice/screening on health risks for the mother (n = 3 indicators), counseling on family planning/return to fertility for the mother (n = 8 indicators), and postnatal care for the newborn (n = 5 indicators). Of 23 postnatal care indicators attempted, 18 indicators in Kenya and 19 in Swaziland had adequate sample size for validation (**Table 3** and **Table 4**). Of these, 12 indicators in Kenya and five of 19 in Swaziland met criteria for both high individual and population-level accuracy. Two indicators met the criteria in both countries. These were: whether during the consultation the provider conducted a breast exam or examined the mother’s abdomen (**Table 5**). An additional four indicators met moderate criteria in both countries: whether the provider checked the mother’s blood pressure, performed a vaginal check for the mother, discussed the benefits of birth spacing, or immunized the baby.

**Table 3:** Postnatal care validation results: Kenya, pooled data collection rounds 2009-2012

Indicator	Total Matched N*		Observer Report: Prevalence of Intervention (%)	Woman’s Self Report: Prevalence of Intervention (%)	Sensitivity (%)	Specificity (%)	Estimated Survey Prevalence† (%)		IF‡		AUC (95% CI)	Met AUC & IF?
**Physical examination of the mother:**												
Blood pressure check		319	41.1	39.8	78.7 (70.6, 85.5)	83.9 (77.9, 88.8)		41.9	**1.02**	**0.813 (0.769, 0.857)**		Yes
Breast exam		316	31.0	29.1	75.0 (64.9, 83.4)	87.1 (81.9, 91.2)		32.2	**1.04**	**0.810 (0.761, 0.86)**		Yes
Examine abdomen		320	33.1	24.4	83.3 (73.2, 90.8)	83.1 (77.7, 87.6)		38.9	**1.18**	**0.832 (0.784, 0.88)**		Yes
Examine vagina		309	20.4	8.1	56.0 (34.9, 75.6)	82.7 (77.8, 87.0)		25.2	**1.23**	*0.694 (0.592, 0.795)*		No
Screen for cervical cancer		NA	NA	NA	NA	NA		NA	NA	NA		NA
Check anemia§		310	29.0	32.3	60.0 (49.7, 69.7)	85.7 (80.2, 90.1)		27.5	**0.95**	0.729 (0.675, 0.782)		Yes
**Advice/screening on health risks for mother:**												
Ask about excessive bleeding		316	40.8	31.3	83.8 (75.1, 90.5)	78.8 (72.8, 84)		46.8	**1.15**	**0.813 (0.768, 0.859)**		Yes
Discuss danger signs after birth||		318	29.2	37.1	61.0 (51.6, 69.9)	89.5 (84.4, 93.4)		25.3	**0.86**	**0.753 (0.704, 0.802)**		Yes
Discuss STIs or HIV/AIDS		321	34.6	35.8	68.7 (59.4, 77)	84.5 (78.8, 89.1)		33.9	**0.98**	**0.766 (0.717, 0.815)**		Yes
**Counseling on family planning/return to fertility:**												
Discuss how soon after delivery a woman can get pregnant		535	25.8	30.5	48.5 (40.6, 56.4)	84.1 (80, 87.7)		24.3	**0.94**	*0.663 (0.620, 0.706)*		No
Discuss return to fertility		543	22.8	30.8	48.5 (40.7, 56.3)	88.6 (84.9, 91.6)		19.9	**0.87**	*0.685 (0.644, 0.727)*		No
Discuss benefits of birth spacing		320	30.3	44.4	55.6 (47.1, 64)	89.9 (84.5, 93.9)		23.9	**0.79**	**0.728 (0.681, 0.774)**		Yes
Discuss return to sexual activity		540	15.9	17.0	50.0 (39.4, 60.6)	91.1 (88.0, 93.5)		15.5	**0.97**	**0.705 (0.652, 0.758)**		Yes
Discuss family planning (FP) method (incl. natural methods)¶		355	65.6	60.8	93.1 (88.8, 96.1)	77 (69.1, 83.7)		69.0	**1.05**	**0.850 (0.811, 0.889)**		Yes
Receive any modern FP method**		NA	NA	NA	NA	NA		NA	NA	NA		NA
Explains advan/disad of chosen FP method		NA	NA	NA	NA	NA		NA	NA	NA		NA
**Postnatal care for the newborn:**												
Discuss breastfeeding/feeding for baby		529	62.2	66.9	79.4 (74.8, 83.5)	72.6 (65.3, 79.0)		59.7	**0.96**	**0.760 (0.72, 0.799)**		Yes
Examine baby (undressed)		307	74.6	60.3	82.7 (76.5, 87.9)	37.7 (29.1, 46.9)		77.5	**1.04**	*0.602 (0.551, 0.653)*		No
Weigh the baby		307	92.2	85.3	96.6 (93.6, 98.4)	33.3 (20.0, 49.0)		94.2	**1.02**	*0.649 (0.579, 0.72)*		No
Immunize baby		455	87.7	87.0	96.2 (93.8, 97.9)	69.5 (56.1, 80.8)		88.1	**1.00**	**0.829 (0.769, 0.889)**		Yes
Gave information on baby’s sickness signs††		355	61.4	46.2	81.7 (74.9, 87.3)	56 (48.7, 63.2)		67.1	**1.09**	*0.689 (0.643, 0.735)*		No

CI – confidence interval, AUC – area under the receiver operating curve, IF – inflation factor

NA = estimate suppressed due to low sample size (n <5 per cell of 2 by 2 table). Numbers in bold: Meets criteria for high individual level accuracy (AUC>0.70) and/or low population-level bias (0.75<IF<1.25). Numbers in italics: Meets criteria for moderate individual-level accuracy (0.60≤AUC≤0.70).

*Sample sizes vary by indicator as not all questions were asked in each survey round and due to participant non-response.

†Estimated survey prevalence calculated using equation: P × (SE + SP – 1) – (1 – SP), where P = Observed prevalence, SE = Sensitivity (proportion of true positives correctly classified by clients), SP = Specificity (proportion of true negatives correctly classified by clients).

‡Inflation Factor (IF) = Estimated survey-based prevalence / Observed prevalence.

§Anemia checked by referral for blood test or by checking woman’s pallor (examine finger nails or lower eyelid).

||Danger signs for mother include: foul smelling discharge, fever, bleeding, broken scars, painful nipples, painful breasts.

¶Natural family planning methods include abstinence and lactational amenorrhea.

**Modern methods include injectables, pill, IUD, implant, progestin-only (breastfeeding mothers), condom use and sterilization.

††Danger signs for baby include difficulties feeding, difficulties breathing, body feels hot/cold or jaundice.

**Table 4:** Postnatal care validation results: Swaziland, Pooled Data Collection Rounds 2009-2012

Indicator	Total Matched N*	Observer Report: Prevalence of Intervention (%)	Woman’s Self-Report: Prevalence of Intervention (%)	Sensitivity (%)	Specificity (%)	Est. Survey Prev.† (%)	IF‡	AUC (95% CI)	Met AUC & IF?
**Physical examination of the mother:**									
Blood pressure check	114	74.6	74.6	83.5 (73.9, 90.7)	51.7 (32.5, 70.6)	74.6	**1.00**	0.676 (0.58, 0.78)	No
Breast exam	117	72.6	74.4	86.2 (77.1, 92.7)	66.7 (47.2, 82.7)	71.7	**0.99**	**0.764 (0.67, 0.86)**	Yes
Examine abdomen	112	64.3	75.0	75.0 (64.4, 83.8)	67.9 (47.6, 84.1)	59.7	**0.93**	**0.714 (0.61, 0.81)**	Yes
Examine vagina	112	71.4	74.1	83.1 (73.3, 90.5)	62.1 (42.3, 79.3)	70.2	**0.98**	**0.726 (0.63, 0.82)**	Yes
Screen for cervical cancer	121	48.8	17.4	81.0 (58.1, 94.6)	58.0 (47.7, 67.8)	61.0	1.25	*0.695 (0.60, 0.79)*	No
Check anemia§	113	38.9	70.8	42.5 (31.5, 54.1)	69.7 (51.3, 84.4)	35.0	**0.90**	0.561 (0.46, 0.66)	No
**Advice/screening on health risks for mother:**									
Ask about excessive bleeding	115	61.7	35.7	65.9 (49.4, 79.9)	40.5 (29.3, 52.6)	63.4	**1.03**	0.532 (0.44, 0.62)	No
Discuss danger signs after birth||	115	49.6	52.2	58.3 (44.9, 70.9)	60.0 (45.9, 73.0)	49.1	**0.99**	0.592 (0.50, 0.68)	No
Discuss STIs or HIV/AIDS	113	63.7	49.6	64.3 (50.4, 76.6)	36.8 (24.4, 50.7)	63.9	**1.00**	0.506 (0.42, 0.60)	No
Counseling on family planning/ return to fertility:									
Discuss how soon after delivery a woman can get pregnant	170	25.9	50.6	32.6 (22.8, 43.5)	81 (70.9, 88.7)	22.5	**0.87**	0.568 (0.5, 0.63)	No
Discuss return to fertility	169	16.6	50.3	23.5 (15.0, 34.0)	90.5 (82.1, 95.8)	11.8	0.71	0.57 (0.51, 0.63)	No
Discuss benefits of birth spacing	190	35.3	66.3	42.1 (33.3, 51.2)	78.1 (66, 87.5)	29.0	**0.82**	*0.601 (0.53, 0.67)*	No
Discuss return to sexual activity	172	24.4	49.4	38.8 (28.4, 50.0)	89.7 (81.3, 95.2)	17.3	0.71	*0.642 (0.58, 0.7)*	No
Discuss family planning (FP) method (incl. natural methods)¶	189	78.8	69.8	78.0 (70, 84.8)	19.3 (10, 31.9)	78.6	**1.00**	0.487 (0.42, 0.55)	No
Receive any modern FP method**	217	65.0	74.2	79.5 (72.4, 85.5)	76.8 (63.6, 87)	59.8	**0.92**	**0.781 (0.72, 0.85)**	Yes
Explains advan/disadv of chosen FP method	231	41.1	46.8	53.7 (43.8, 63.3)	69.9 (61, 77.9)	39.8	**0.97**	*0.618 (0.56, 0.68)*	No
**Postnatal care for newborn:**									
Discuss breastfeeding/feeding for baby	279	83.9	95.7	NA	NA	NA	NA	NA	NA
Examine baby (undressed)	118	92.4	80.5	95.8 (89.6, 98.8)	21.7 (7.5, 43.7)	94.5	**1.02**	0.588 (0.50, 0.68)	No
Weigh the baby	117	96.6	96.6	NA	NA	NA	NA	NA	NA
Immunize baby	309	87.1	81.2	93.2 (89.4, 96.0)	39.7 (27, 53.4)	89.0	**1.02**	*0.664 (0.60, 0.73)*	No
Gave information on baby’s sickness signs††	166	59.0	67.5	60.7 (51.0, 69.8)	44.4 (30.9, 58.6)	58.6	**0.99**	0.526 (0.44, 0.61)	No

NA – estimate suppressed due to low sample size (n <5 per cell of 2 by 2 table). Numbers in bold: Meets criteria for high individual level accuracy (AUC>0.70) and/or low population-level bias (0.75<IF<1.25). Numbers in italics: Meets criteria for moderate individual-level accuracy (0.60≤AUC≤0.70).

*Sample sizes vary by indicator as not all questions were asked in each survey round and due to participant non-response.

†Estimated survey prevalence calculated using equation: P × (SE + SP – 1) – (1 – SP), where P = Observed prevalence, SE = Sensitivity (proportion of true positives correctly classified by clients), SP = Specificity (proportion of true negatives correctly classified by clients).

‡Inflation Factor (IF) = Estimated survey-based prevalence / Observed prevalence.

§Anemia checked by referral for blood test or by checking woman’s pallor (examine finger nails or lower eyelid).

||Danger signs for mother include: foul smelling discharge, fever, bleeding, broken scars, painful nipples, painful breasts.

¶Natural family planning methods include abstinence and lactational amenorrhea.

**Modern methods include injectables, pill, IUD, implant, progestin-only (breastfeeding mothers), condom use and sterilization.

††Danger signs for baby include difficulties feeding, difficulties breathing, body feels hot/cold or jaundice.

**Table 5:** Postnatal care indicator summary results by country*

Indicator	Kenya		Swaziland	
**AUC**	**IF**	**AUC**	**IF**
Blood pressure check	Y	Y		Y
Breast exam	Y	Y	Y	Y
Examine abdomen	Y	Y	Y	Y
Examine vagina		Y	Y	Y
Screen for cervical cancer	NA	NA		
Check anemia†	Y	Y		Y
Ask about excessive bleeding	Y	Y		Y
Discuss danger signs after birth‡	Y	Y		Y
Discuss STIs or HIV/AIDS	Y	Y		Y
Discuss how soon after delivery a woman can get pregnant		Y		Y
Discuss return to fertility		Y		
Discuss benefits of birth spacing		Y		Y
Discuss return to sexual activity	Y	Y		
Discuss family planning (FP) method (incl. natural methods)§	Y	Y		Y
Receive any modern FP method||	NA	NA	Y	Y
Explains advantages/disadvantages of chosen FP method	NA	NA		Y
Discuss breastfeeding/feeding for baby	Y	Y	NA	NA
Examine baby (undressed)		Y		Y
Weigh the baby		Y	NA	NA
Immunize baby	Y	Y		Y
Gave information on baby’s sickness signs¶		Y		Y

AUC – area under receiver operating curve, IF – inflation factor

*Y – indicates validity criteria were met, NA – insufficient sample size in country to assess indicator. Blank indicates there was sufficient sample size and the criterion were not met.

†Anemia checked by referral for blood test or by checking woman’s pallor (examine finger nails or lower eyelid).

‡Danger signs for mother include: foul smelling discharge, fever, bleeding, broken scars, painful nipples, painful breasts.

§Natural family planning methods include abstinence and lactational amenorrhea.

||Modern methods include: injectables, pill, IUD, implant, progestin-only (breastfeeding mothers), condom use and sterilization.

¶Danger signs for baby include: difficulties feeding, difficulties breathing, body feels hot/cold or jaundice.

In both Kenya and Swaziland, the subset of indicators related to the physical examination of the mother demonstrated high individual-level accuracy relative to other phases of the postnatal visit. Specifically, of the five physical examination indicators for the mother with sufficient sample size in Kenya, four indicators had high individual-level accuracy (AUC>0.70) (blood pressure check, breast exam, abdominal exam and check for anemia). Of the six indicators related to the physical examination of the mother in Swaziland, three had high individual-level accuracy (breast exam, abdominal exam, vaginal exam) (AUC>0.70). One exception was that low individual-level accuracy was observed for the check for anemia in Swaziland (AUC = 0.56, 95% CI = 0.46, 0.66), which may result from the fact that women were not always aware of the purpose of examination.

Of indicators related to advice or screening on health risks for the mother (whether the provider asked about excessive bleeding, discussed postpartum danger signs with the mother, or discussed STIs or HIV/AIDS), all three indicators in Kenya met criteria for individual-level accuracy (AUC>0.70) and low population-level bias (0.75<IF<1.25). In contrast, in Swaziland, no indicator met criteria for individual-level accuracy, although criteria for population-level measurement were met. These results imply that women’s false positive and false negative reports cancel out at the aggregate level and indicate the measure may be suitable for measurement at the population-level only in Swaziland.

In terms of indicators related to women’s return to fertility or use of family planning, it was possible to validate six indicators in Kenya and eight in Swaziland. All indicators in Kenya met acceptability criteria for individual and population-level accuracy. Two indicators with insufficient sample size for validation in Kenya – whether the woman received any modern method of contraception and whether the provider discussed the advantages or disadvantages of the chosen family planning method – met both criteria in Swaziland. Only one additional indicator in Swaziland met both acceptability criteria – whether the provider discussed the benefits of birth spacing with the mother. Particularly low individual-level accuracy was observed for whether the provider discussed any family planning method in Swaziland (AUC = 0.49, 95% CI = 0.42, 0.55) likely due to the low specificity of the indicator (19%).

We assessed five indicators related to care for the baby during the postnatal visit. These included: whether the baby was examined (undressed), weighed, and immunized, and whether breastfeeding/feeding the baby and danger signs related to the baby’s health were discussed. Of these, there was insufficient variation in reported outcomes to validate two indicators in Swaziland – whether the provider discussed breastfeeding/feeding for the baby or weighed the baby. Two indicators met both acceptability criteria in Kenya – whether the provider discussed breastfeeding/feeding for the baby or gave information on sickness signs for the baby. No indicator met both acceptability criteria in Swaziland. Whether the provider examined the baby undressed had low specificity (22%) and the overall AUC was low (AUC = 0.59, 95% CI = 0.5, 0.68). Low specificity could result from ambiguous question wording for this indicator in the exit interview with regard to whether the baby was unclothed during the examination (**Table 1**).

**Figure 1** and **Figure 2** illustrate intervention coverage that would be estimated in a household survey using the sensitivity and specificity of women’s recall observed in Kenya (blue line) and Swaziland (red line) across actual intervention coverage levels ranging from 0 to 100%. The black line represents perfect reporting accuracy (100% sensitivity and specificity). **Figure 1** uses the example of whether the provider discussed danger signs for the mother after birth. In low coverage areas (<20%), the estimated coverage of this intervention is substantially overestimated using the properties of women’s recall in Swaziland, while only slightly overestimated using those of women in Kenya. However, in high coverage areas (>80%) this indicator is substantially underestimated in both populations. These results are observed given the high specificity of the indicator in Kenya (90%) relative to Swaziland (60%), and only moderate sensitivity in both countries (~ 60%). The estimated coverage of infant immunization (**Figure 2**), on the other hand, closely approximates the true coverage in settings where the practice is common (>80% coverage) given the high sensitivity of women’s recall in Kenya and Swaziland (~ 95%). However, the estimated coverage of infant immunization would be greatly overestimated in low coverage settings of both countries, particularly in Swaziland where the specificity of women’s recall was relatively low (40%).

### Women’s reporting accuracy by covariates

The sensitivity, specificity and accuracy of recalling whether postnatal care interventions were received, stratified by women’s sociodemographic characteristics, are shown in Tables S1-S4 in **Online Supplementary Document(Online Supplementary Document)**. Across the four indicators assessed (whether the provider discussed breastfeeding/feeding for the baby, discussed postpartum danger signs for the mother after birth, weighed the baby or immunized the baby), we identified no overall pattern in the attributes of women most likely to recall PNC interventions accurately across indicators. Table S5 in **Online Supplementary Document(Online Supplementary Document)** presents covariate-adjusted receiver operating curve coefficients for each of the four outcomes. These data indicate that facility, year of data collection and age of client did not significantly influence the discrimination accuracy of women’s recall of any of the four interventions. In contrast to what would be expected, higher education (secondary or higher) was observed to negatively influence women’s reporting of whether the provider discussed breastfeeding/infant feeding (β = -0.28, 95% CI = -0.49, -0.07). Having an older aged infant negatively influenced women’s recall of whether the provider discussed postpartum danger signs for the mother (β = -0.19, 95% CI = -0.35, -0.03), but positively influenced women’s recall of whether the baby was immunized (β = 0.25, 95% CI = 0.10, 0.40). These discrepancies may be explained by the timing of when interventions occurred in relation to the age of the infant. Finally, higher parity was observed to positively influence women’s recall of whether the infant was weighed during the consultation (β = 0.11, 95% CI = 0.02, 1.8), which could be attributed to the fact that mothers with prior births are more aware of what interventions to expect during the visit.

## DISCUSSION

Both the DHS and MICS programs currently collect data on the occurrence of PNC health visits for the mother and newborn. However, these data provide limited information on the content or quality of postnatal care. Currently, no questions included in the DHS or MICS relate to the content of care mothers receive during their postnatal visit. Only recently have more detailed questions related to newborn care during the postnatal period been included in DHS and MICS (ie, whether during the first two days any health care provider examined the cord, measured the infant’s temperature, counseled the mother on danger signs for newborns, counseled the mother on breastfeeding or observed breastfeeding). While these questions expand upon longstanding questions in both survey programs related to whether the mother breastfed the infant in the first hour of birth, the receipt of immunizations, and infant weight, they have yet to be empirically validated.

Results of this study suggest that women are able to report accurately on multiple aspects of care received during the postnatal period. Specifically, of 18 indicators analyzed in Kenya, 12 indicators (ten related to maternal care and two related to newborn care) met criteria for individual and population-level reporting accuracy. Of 19 assessed indicators in Swaziland, five indicators (all related to maternal care and none related to newborn care) met both acceptability criteria. Two indicators met high acceptability criteria in both Kenya and Swaziland: whether the provider performed a breast exam or an abdominal exam for the mother.

This study also informs the validity of four newborn PNC indicators currently included in the DHS and MICS: counseling on breastfeeding, counseling on infant danger signs, weighing of the baby, and receipt of child immunizations. We show that one indicator– receipt of child immunizations – was reported with moderate or higher accuracy in both countries. The indicators of whether the infant was weighed and whether the mother was counseled on breastfeeding also met criteria for moderate and high accuracy, respectively, in Kenya. However, neither indicator could be assessed in Swaziland due to low variation in the data. Specifically, the practices were both highly prevalent and highly reported by women (high sensitivity). While this suggests these indicators may be validly reported, they should be assessed in settings where they are not universal to better inform indicator specificity (true negative results). The final indicator, whether the mother was counseled on danger signs for the newborn, did not meet criteria for individual-level accuracy in either country. However, while the intention of the indicator was to ask women about danger signs for the newborn, the question wording may be subject to misinterpretation among women (**Table 1**), which could contribute to lower reporting accuracy. With respect to content of care indicators for maternal PNC, results from this study suggest that indicators that reflect physical examination of the mother are generally accurately reported. Not only do both indicators that met high acceptability criteria in both countries (breast and abdominal exam) relate to physical examination, but two additional indicators met moderate or higher criteria in both countries: whether the provider performed a blood pressure check or vaginal check for the mother. A third indicator – whether the provider discussed the benefits of birth spacing, also met moderate or higher criteria in both countries. Taken together, these results suggest that additional content of care indicators related to the newborn and for the mother can be accurately reported. Indicators of the mother’s physical examination may be particularly informative given the potential to inform health risks to the mother.

In contrast to earlier validation research which has examined women’s reporting accuracy on indicators related to the immediate postnatal period (within the first hour of birth) in LMIC contexts, results from this study provide encouraging evidence that women are generally able to more accurately report on care received in the postnatal period (from 24 hours to 10 weeks after birth). For example, a study of the same design in Kenya where women were interviewed at hospital discharge and were also followed up again at home approximately one year following delivery, found that only one indicator related to the content of immediate postnatal care met the same criteria for individual and population-level accuracy at both time points – whether the newborn was low birthweight (<2500 g). Another study among women in Mozambique found support for the validity of an indicator on whether or not the newborn was placed naked directly against the mothers’ skin following delivery [27]. While this finding was also observed in the Kenya study at hospital discharge, validity declined at 13-15 months postpartum and did not meet validation criteria at delayed follow-up [28,30]. An additional immediate postnatal indicator that met acceptability criteria at hospital discharge but where validity declined upon re-interview 13-15 months later was whether the provider took the woman’s temperature in the first physical exam in the facility following delivery [28,30]. Finally, a study in China also assessed women’s ability to report on the content of postnatal care indicators related to physical examination of the mother and provision of family planning counseling relative to an electronic record reference standard [14]. The overall AUC among indicators of various aspects of women’s physical examination were lower than those observed in the present study, due primarily to lower specificity. In both studies, measures of population-level accuracy (the TAP ratio reported by Liu et al., is the mathematical equivalent to the IF calculated in the present study) were close to 1, indicating low population-level bias [14]. Discrepancies between the present study and Liu et al. may be attributable to differences in the reference standard (direct observation vs. electronic records) and recall time period of the woman (exit interview vs two to five-year recall period).

Results from this study also demonstrate that not all indicators work well in both settings. Reasons for reporting discrepancies between countries could result from variations in interviewer or observer quality due to differences in training or supervision between countries, or differences in provider services that more clearly addressed particular areas of health. For example, this study used data collected as part of an intervention to integrate HIV and family planning care with PNC. As part of the intervention, HIV-related care was more strongly emphasized in Swaziland, where HIV prevalence among pregnant women is extremely high [31], while family planning counseling was more heavily emphasized in Kenya. If providers more clearly explained the procedures for certain aspects of care, women may have better recalled whether these interventions were received. Covariate analysis of the Kenya data did not provide strong evidence to suggest that variation in women’s sociodemographic characteristics, the facility where care was received, or year of interview, explained differences in recall accuracy across aspects of care. To better understand differences in reporting accuracy between countries, additional research in a range of settings is warranted.

There are several limitations to the present research. First, while it is a strength that this study utilizes direct observation by a third-party observer as the reference standard, it was only possible to observe women who visited health facilities for postnatal care. Therefore, our results reflect the reporting accuracy of women who seek facility-based care. Despite limitations in the generalizability of reported results to women who receive home-based or no postnatal care, these findings are an important first step to understanding what elements of postnatal care women can report on with accuracy. In addition, the exit interview that occurred immediately after the visit does not exactly replicate that circumstances under which women would be asked to report in a household survey. On average, interviews in the DHS and MICS would take place approximately 2.5 years after the PNC visit occurred. A previous study in Kenya that examined labor, delivery, and immediate postpartum indicators did not find systematic evidence of a deterioration in reporting quality across all indicators [30], but a similar examination of postnatal care indicators has not been done.

Another limitation in validation research of this and similar design is that it is difficult to distinguish between true indicator properties (ie, women’s knowledge or recall of the intervention) and noise introduced by measurement error (e.g., misunderstanding or poor-quality interview). Additionally, the on-going Integra Initiative (which the present analysis draws upon for secondary analysis) or the presence of observers in the facilities could have influenced provider behavior. For example, providers may have more clearly explained their actions than those in a typical health facility setting, which could reduce the ability to generalize findings to settings with lower quality care. Further, it is difficult to understand the degree to which variation in reporting accuracy is attributable to measurement error as opposed to other factors such as participant characteristics. Overcoming such limitations draws attention to the need to improve upon the design of validation studies, potentially by supplementing quantitative findings with qualitative research to inform how women understand questions and specific terminology. Finally, the sample size in Swaziland was lower than anticipated due to the inability to match all cases. However, we believe data are missing at random which limits the potential for systematic bias among included cases. Finally, we did not perform a power analysis for each covariate strata; covariate results should be interpreted with caution.

## CONCLUSIONS

The postnatal period is a high-risk period for maternal and newborn health. Despite this and the recommendations of several maternal and newborn health agendas, including: the Global Strategy 2.0 Measurement Framework, the Sustainable Development Goals, the Every Newborn Action Plan (ENAP) and Ending Preventable Maternal Mortality (EPMM), few indicators included in population based surveys collect information on the content of postnatal care. Those that do relate to newborn care only. No existing indicators in household survey programs such as the DHS or MICS currently monitor the content of care for the mother that is received during a postnatal visit. Our findings suggest women are able to accurately report on multiple aspects of care received during a postnatal visit. Further development and inclusion of postnatal care indicators, particularly for mothers, is warranted in population-level surveillance in order to improve the quality and coverage of maternal and newborn care.

## References

[1] Kassebaum NJ, Bertozzi-Villa A, Coggeshall MS, Shackelford KA, Steiner C, Heuton KR (2014). Global, regional, and national levels and causes of maternal mortality during 1990-2013: A systematic analysis for the Global Burden of Disease Study 2013.. Lancet.

[2] Say L, Chou D, Gemmill A, Tunçalp Ö, Moller A-B, Daniels J (2014). Global causes of maternal death: a WHO systematic analysis.. Lancet Glob Health.

[3] Partnership for Maternal. Newborn and Child health. Opportunities for Africa's Newborns. Practical data, policy and programmatic support for newborn care in Africa. 2006. Available: http://www.who.int/pmnch/media/publications/oanfullreport.pdf. Accessed: July 25, 2016.

[4] Child Mortality Collaborators GBD (2016). Global, regional, national, and selected subnational levels of stillbirths, neonatal, infant, and under-5 mortality, 1980-2015: A systematic analysis for the Global Burden of Disease Study 2015.. Lancet.

[5] Horta BL, Victora CG. Short-term effects of breastfeeding. A systematic review on the benefits of breastfeeding on diarrhoea and pneumonia mortality. Geneva: World Health Organization; 2013. Available: http://apps.who.int/iris/bitstream/10665/95585/1/9789241506120_eng.pdf. Accessed: August 4, 2016.

[6] Paintsil E, Andiman WA (2009). Update on successes and challenges regarding mother-to-child transmission of HIV.. Curr Opin Pediatr.

[7] Tripathi V, Stanton C, Strobino D, Bartlett L (2015). Development and validation of an index to measure the quality of facility-based labor and delivery care processes in sub-Saharan Africa.. PLOS ONE.

[8] Graham WJ, Bell JS, Bullough CHW. Can skilled attendance at delivery reduce maternal mortality in developing countries? Studies in Health Services Organisation and Policy. 2001. Available: http://www.jsieurope.org/safem/collect/safem/pdf/s2934e/s2934e.pdf. Accessed: August 12, 2016.

[9] Hodgins S (2013). Achieving better maternal and newborn outcomes: coherent strategy and pragmatic, tailored implementation.. Glob Health Sci Pract.

[10] Moran AC, Kerber K, Sitrin D, Guenther T, Morrissey CS, Newby H (2013). Measuring coverage in MNCH: Indicators for global tracking of newborn care.. PLoS Med.

[11] Newborn Health Indicators Working Group. Meeting Report 2014. Healthy Newborn Network. 2014 Jan 23. Available: http://www.healthynewbornnetwork.org/resource/meeting-report-newborn-health-indicators-working-group-meeting-january-23-24-2014/. Accessed: July 28, 2016.

[12] The DHS ProgramAvailable: http://dhsprogram.com/publications/publication-dhsq7-dhs-questionnaires-and-manuals.cfm. Accessed: 9 January 2017.

[13] World Health Organization. Recommendations on Postnatal Care of the Mother and Newborn. 2013. Available: http://apps.who.int/iris/bitstream/10665/97603/1/9789241506649_eng.pdf. Accessed: July 31, 2016. 24624481

[14] Liu L, Li M, Yang L, Ju L, Tan B, Walker N (2013). Measuring coverage in MNCH: A validation study linking population survey derived coverage to maternal, newborn, and child health care records in rural China.. PLoS One.

[15] Hill Z, Okyere E, Wickenden M, Tawiah-Agyemang C (2015). What can we learn about postnatal care in Ghana if we ask the right questions? A qualitative study.. Glob Health Action.

[16] Yoder PS, Rosato M, Mahmud R, Fort A, Rahman F, Armstrong A, et al. Women's recall of delivery and neonatal care in Bangladesh and Malawi: A study of terms, concepts, and survey questions. 2010. DHS Qualitative Research Studies No. 17. Calverton, MD, USA: ICF Macro. Available: http://dhsprogram.com/publications/publication-qrs17-qualitative-research-studies.cfm. Accessed: October 25, 2015.

[17] Warren CE, Mayhew SH, Vassall A, Kimani JK, Church K, Obure CD (2012). Study protocol for the Integra Initiative to assess the benefits and costs of integrating sexual and reproductive health and HIV services in Kenya and Swaziland.. BMC Public Health.

[18] Kenya National Bureau of Statistics, Ministry of Health Kenya, National AIDS Control Council, Kenya Medical Research Institute, National Council for Population and Development Kenya, ICF International. Demographic and Health Survey: Kenya 2014. Nairobi, Kenya; Rockville, Maryland; 2015. Available: http://dhsprogram.com/publications/publication-fr308-dhs-final-reports.cfm. Accessed: April 16, 2016.

[19] Central Statistical Office Swaziland. Macro International. Swaziland Demographic and Health Survey 2006-07. Mbabane, Swaziland; Calverton MD, USA; 2008. Available: http://dhsprogram.com/pubs/pdf/fr202/fr202.pdf. Accessed: April 17, 2016.

[20] Buderer NM (1996). Statistical methodology: I. Incorporating the prevalence of disease into the sample size calculation for sensitivity and specificity.. Acad Emerg Med.

[21] Robin X, Turck N, Hainard A, Tiberti N, Lisacek F, Sanchez J-C (2011). pROC: an open-source package for R and S+ to analyze and compare ROC curves.. BMC Bioinformatics.

[22] Eng J (2005). Receiver operating characteristic analysis: a primer.. Acad Radiol.

[23] Metz CE (1989). Some practical issues of experimental design and data analysis in radiological ROC studies.. Invest Radiol.

[24] Hanley JA, McNeil BJ (1982). The meaning and use of the area under a receiver operating characteristic (ROC) curve.. Radiology.

[25] Vecchio TJ (1966). Predictive value of a single diagnostic test in unselected populations.. N Engl J Med.

[26] Campbell H, Biloglav Z, Rudan I (2008). Reducing bias from test misclassification in burden of disease studies: use of test to actual positive ratio–new test parameter.. Croat Med J.

[27] Stanton CK, Rawlins B, Drake M, Anjos dos M, Cantor D, Chongo L (2013). Measuring coverage in MNCH: Testing the validity of women’s self-report of key maternal and newborn health Interventions during the peripartum period in Mozambique.. PLoS One.

[28] Blanc AK, Warren C, McCarthy K, Kimani J, Ndwiga C (2016). RamaRao S. Assessing the validity of indicators of the quality of maternal and newborn health care in Kenya.. J Glob Health.

[29] Janes H, Longton G, Pepe M (2009). Accommodating covariates in ROC analysis.. Stata J.

[30] McCarthy KJ, Blanc AK, Warren CE, Kimani J, Mdawida B, Ndwidga C (2016). Can surveys of women accurately track indicators of maternal and newborn care? A validity and reliability study in Kenya.. J Glob Health.

[31] Warren CE, Abuya T, Askew I (2013). Family planning practices and pregnancy intentions among HIV-positive and HIV-negative postpartum women in Swaziland: a cross sectional survey.. BMC Pregnancy Childbirth.

