# Supplementary material for: Women’s recall of maternal and newborn interventions received in the postnatal period: a validity study in Kenya and Swaziland
Source: J Glob Health. 2018 May 12;8(1):010605. doi: 10.7189/jogh.08.010605 (PMC5983915; doi:10.7189/jogh.08.010605)
Supplement: Online Supplementary Document [file jogh-08-010605-s001.pdf]

Online Supplementary Document

McCarthy et al. Women's recall of maternal and newborn interventions received in the postnatal period: a validity study in Kenya and Swaziland

J Glob Health 2018;8:010605

Supplemental Table 1: Accuracy of women's recall of whether provider discussed breastfeeding/feeding for the baby by interview and socio-demographic characteristics, Kenya, 2009 - 2012

	Sensitivity (%) (95%CI)	Specificity (%) (95%CI)	AUC (95%CI)	Sample Size
Survey Round				
R0 2009	84.3 (76.4,90.5)	87.7 (79.9, 93.3)	0.860 (0.815, 0.906)	221
R2 2011	85.1 (76.7,91.4)	48.0 (27.8, 68.7)	0.666 (0.560, 0.772)	126
R3 2012	71.0 (62.7,78.4)	50.0 (34.6, 65.4)	0.605 (0.521, 0.689)	182
Age of Client				
15-24	82.2 (75.2,88)	64.6 (53.3, 74.9)	0.734 (0.674, 0.795)	234
25-34	78.8 (71.6,85.0)	81.3 (70.7, 89.4)	0.801 (0.746, 0.856)	231
35-44	76.9 (60.7,88.9)	72.2 (46.5, 90.3)	0.746 (0.620, 0.872)	57
Age of Baby				
<2wks	80.2 (70.6,87.8)	47.4 (24.4, 71.1)	0.638 (0.515, 0.760)	110
2-4wks	76.3 (65.2,85.3)	75.0 (58.8, 87.3)	0.757 (0.673, 0.840)	116
5-6wks	80.9 (73.8,86.8)	75.6 (64.9, 84.4)	0.783 (0.726, 0.839)	234
7-10wks	79.4 (62.1,91.3)	76.5 (58.8, 89.3)	0.779 (0.679, 0.879)	68
Prior Parity				
1	82.0 (73.1,89.0)	72.7 (59, 83.9)	0.774 (0.703, 0.844)	155
2	77.8 (67.2,86.3)	71.8 (55.1, 85)	0.748 (0.663, 0.833)	120
3	80.0 (68.2,88.9)	72.7 (54.5, 86.7)	0.764 (0.672, 0.855)	98
4+	80.2 (71.1,87.5)	75.0 (59.7, 86.8)	0.776 (0.700, 0.852)	145
Education Level				
Less than secondary	84.5 (76.6,90.5)	87.0 (78.8, 92.9)	0.778 (0.736, 0.820)	429
Secondary or more	70.4 (58.4,80.7)	65.5 (45.7, 82.1)	0.680 (0.577, 0.783)	100
**Total**	**79.4**	**72.6**	**0.76 (0.72, 0.799)**	**529**

NA= estimate suppressed due to small sample size.

Supplemental Table 2: Accuracy of women's recall of whether provider discussed postpartum danger signs for the mother by interview and socio-demographic characteristics, Kenya, 2009 - 2012

	Sensitivity (%)	Specificity (%)	AUC (95%CI)	Sample Size
Round				
R0 2009	NA	NA	NA	0
R2 2011	63.2 (49.3,75.6)	85.7 (75.3, 92.9)	0.744 (0.669, 0.820)	127
R3 2012	59.0 (45.7,71.4)	91.5 (85.4, 95.7)	0.753 (0.686, 0.819)	191
Age of Client				
15-24	66.1 (53.0,77.7)	90.8 (81.9, 96.2)	0.785 (0.717, 0.852)	138
25-34	55.0 (38.5,70.7)	90.7 (83.1, 95.7)	0.729 (0.645, 0.812)	137
35-44	56.2 (29.9,80.2)	78.3 (56.3, 92.5)	0.673 (0.520, 0.825)	39
Age of Baby				
<2wks	65.5 (51.9,77.5)	85.5 (73.3, 93.5)	0.755 (0.677, 0.832)	113
2-4wks	68.2 (45.1,86.1)	92.4 (83.2, 97.5)	0.803 (0.698, 0.908)	88
5-6wks	52.8 (35.5,69.6)	88.7 (79.0, 95.0)	0.708 (0.617, 0.798)	107
7-10wks	NA	NA	NA	0
Prior Parity				
1	60.0 (43.3,75.1)	91.1 (80.4, 97.0)	0.755 (0.670, 0.841)	96
2	77.8 (52.4,93.6)	88.5 (76.6, 95.6)	0.831 (0.723, 0.939)	70
3	65.0 (40.8,84.6)	94.6 (81.8, 99.3)	0.798 (0.685, 0.911)	57
4+	52.6 (35.8,69.0)	82.6 (68.6, 92.2)	0.676 (0.579, 0.774)	84
Education Level				
Less than secondary	64.3 (54,73.7)	89.8 (83.1, 94.4)	0.770 (0.716, 0.825)	225
Secondary or more	45.0 (23.1,68.5)	88.9 (79.3, 95.1)	0.669 (0.552, 0.787)	92
**Total**	**61.0 (51.6,69.9)**	**89.5 (84.4, 93.4)**	**0.753 (0.704, 0.802)**	**238**

NA= estimate suppressed due to small sample size.

Supplemental Table 3: Accuracy of women's recall of whether provider weighed the baby by interview and socio-demographic characteristics, Kenya, 2009 - 2012

	**Sensitivity (%)**	**Specificity (%)**	**AUC (95%CI)**	**Sample Size**
Round				
R0 2009	NA	NA	NA	0
R2 2011	99.0 (94.4,100)	20.7 (08.0, 39.7)	0.598 (0.523, 0.674)	126
R3 2012	95.2 (90.7,97.9)	56.2 (29.9, 80.2)	0.757 (0.630, 0.884)	181
Age of Client				
15-24	96.4 (91.0,99.0)	34.6 (17.2, 55.7)	0.655 (0.560, 0.750)	136
25-34	95.7 (90.2,98.6)	35.7 (12.8, 64.9)	0.657 (0.525, 0.789)	130
35-44	100 (89.4,100)	20.0 (00.5, 71.6)	0.600 (0.404, 0.796)	38
Age of Baby				
<2wks	94.9 (87.4,98.6)	22.6 (09.6, 41.1)	0.587 (0.509, 0.666)	109
2-4wks	93.4 (85.3,97.8)	57.1 (18.4, 90.1)	0.753 (0.553, 0.953)	83
5-6wks	100 (96.3,100)	50.0 (11.8, 88.2)	0.750 (0.531, 0.969)	104
7-10wks	100 (66.4,100)	100 (02.5, 100)	NA	10
Prior Parity				
1	94.8 (87.2,98.6)	27.8 (09.7, 53.5)	0.613 (0.504, 0.722)	95
2	94.8 (85.6,98.9)	33.3 (07.5, 70.1)	0.641 (0.475, 0.807)	67
3	95.7 (85.2,99.5)	57.1 (18.4, 90.1)	0.764 (0.564, 0.964)	53
4+	100 (94.9,100)	27.3 (06.0, 61.0)	0.636 (0.498, 0.774)	81
Education Level				
Less than secondary	97.8 (94.6,99.4)	28.1 (13.7, 46.7)	0.630 (0.550, 0.710)	217
Secondary or more	93.5 (85.5,97.9)	46.2 (19.2, 74.9)	0.698 (0.555, 0.842)	90
**Total**	**96.6 (93.6,98.4)**	**33.3 (20.0, 49.0)**	**0.649 (0.579, 0.72)**	**225**

NA= estimate suppressed due to small sample size.

Supplemental Table 4: Accuracy of women's recall of whether provider immunized the baby by interview and socio-demographic characteristics, Kenya, 2009 - 2012

	Sensitivity (%)	Specificity (%)	AUC (95%CI)	Sample Size
Round				
R0 2009	NA	NA	NA	0
R2 2011	95.7 (89.5,98.8)	69.2 (48.2, 85.7)	0.825 (0.732, 0.918)	120
R3 2012	92.4 (86.7,96.1)	76.7 (57.7, 90.1)	0.845 (0.765, 0.925)	174
Age of Client				
15-24	97.5 (93.8,99.3)	68.8 (50.0, 83.9)	0.831 (0.749, 0.914)	193
25-34	95.7 (91.7,98.1)	77.8 (52.4, 93.6)	0.867 (0.767, 0.967)	203
35-44	93.3 (81.7,98.6)	57.1 (18.4, 90.1)	0.752 (0.551, 0.954)	52
Age of Baby				
<2wks	88.6 (80.1,94.4)	83.3 (58.6, 96.4)	0.860 (0.765, 0.954)	106
2-4wks	94.0 (85.4,98.3)	70.8 (48.9, 87.4)	0.824 (0.727, 0.921)	91
5-6wks	99.5 (97.2,100)	53.8 (25.1, 80.8)	0.767 (0.626, 0.908)	207
7-10wks	100 (92.3,100)	50.0 (06.8, 93.2)	NA	50
Prior Parity				
1	97.3 (92.4,99.4)	75.0 (50.9, 91.3)	0.862 (0.763, 0.96)	132
2	97.6 (91.8,99.7)	64.7 (38.3, 85.8)	0.812 (0.694, 0.93)	102
3	94.7 (86.9,98.5)	75.0 (34.9, 96.8)	0.848 (0.686, 1.00)	83
4+	94.7 (88.9,98)	69.2 (38.6, 90.9)	0.82 (0.688, 0.952)	127
Education Level				
Less than secondary	96.6 (94.0,98.3)	75.0 (57.8, 87.9)	0.858 (0.786, 0.93)	361
Secondary or more	94.4 (86.2,98.4)	60.9 (38.5, 80.3)	0.776 (0.671, 0.882)	94
**Total**	**96.2 (93.8,97.9)**	**69.5 (56.1, 80.8)**	**0.829 (0.769, 0.889)**	**434**

NA= estimate suppressed due to small sample size.

Supplemental Table 5. Covariate Adjusted ROC Regression of Influence of Individual and Interview Characteristics on Women's Ability to Recall Four Postnatal Care Interventions, Adjusting for Correlated Error at Facility Level

	Beta Coefficient	95% CI	Wald Test-statistic	P-value
**Discuss Breastfeeding/ Feeding for Baby** (N=522)				
Facility (df=14)	-0.04	(-0.10, 0.02)	-1.38	0.168
Round (df=2)	-0.18	(-0.40, 0.05)	-1.6	0.109
Age of Client (df 2)	-0.07	(-0.21, 0.06)	-1.07	0.283
Age of Baby (df=3)	0.01	(-0.15, 0.17)	0.13	0.897
Prior Parity (df=3)	-0.01	(-0.12, 0.10)	-0.16	0.874
Education (df=1)	-0.28*	(-0.49, -0.07)	-2.47	0.014
**Discuss Danger Signs for Mother After Birth (**N=318)				
Facility (df=14)	-0.04	(-0.23, 0.15)	-0.43	0.664
Round (df=2)	-0.10	(-0.51, 0.31)	-0.46	0.642
Age of Client (df 2)	-0.15	(-0.47, 0.16)	-0.95	0.34
Age of Baby (df=3)	-0.19*	(-0.35, -0.03)	-2.3	0.021
Prior Parity (df=3)	-0.07	(-0.25, 0.12)	-0.72	0.472
Education (df=1)	-0.45	(-1.21, 0.31)	-1.17	0.243
**Weigh the Baby (**N=296)		N=296		
Facility (df=14)	-0.04	(-0.09, 0.01)	-1.67	0.094
Round (df=2)	-0.25	(0-.49, -0.17)	-2.1	0.036
Age of Client (df 2)	0.07	(-0.13, 0.28)	0.7	0.481
Age of Baby (df=3)	0.17	(-0.01, 0.36)	1.85	0.064
Prior Parity (df=3)	0.11**	(0.02, 1.8)	2.78	0.005
Education (df=1)	-0.28	(-0.70, 0.14)	-1.31	0.191
**Immunize the Baby (**N=294)				
Facility (df=14)	-0.02	(-0.07 - 0.03)	-0.82	0.415
Round (df=2)	-0.17	(-0.56 - 0.22)	-0.87	0.387
Age of Client (df 2)	-0.16	(-0.39 - 0.07)	-1.38	0.168
Age of Baby (df=3)	0.25**	(0.10 - 0.40)	3.36	0.001
Prior Parity (df=3)	-0.09	(-0.26 - 0.08)	-1.08	0.28
Education (df=1)	-0.00	(-0.40 - 0.39)	-0.02	0.988

** p<0.01, * p<0.05
#Adjusted for clustering at facility level
df= degrees of freedom
